# Developmental Sex Differences in Nicotinic Currents of Prefrontal Layer VI Neurons in Mice and Rats

**DOI:** 10.1371/journal.pone.0009261

**Published:** 2010-02-17

**Authors:** Nyresa C. Alves, Craig D. C. Bailey, Raad Nashmi, Evelyn K. Lambe

**Affiliations:** 1 Department of Physiology, University of Toronto, Toronto, Ontario, Canada; 2 Department of Biology, University of Victoria, Victoria, British Columbia, Canada; 3 Department of Obstetrics and Gynaecology, University of Toronto, Toronto, Ontario, Canada; University of Queensland, Australia

## Abstract

**Background:**

There is a large sex difference in the prevalence of attention deficit disorder; yet, relatively little is known about sex differences in the development of prefrontal attention circuitry. In male rats, nicotinic acetylcholine receptors excite corticothalamic neurons in layer VI, which are thought to play an important role in attention by gating the sensitivity of thalamic neurons to incoming stimuli. These nicotinic currents in male rats are significantly larger during the first postnatal month when prefrontal circuitry is maturing. The present study was undertaken to investigate whether there are sex differences in the nicotinic currents in prefrontal layer VI neurons during development.

**Methodology/Principal Findings:**

Using whole cell recording in prefrontal brain slice, we examined the inward currents elicited by nicotinic stimulation in male and female rats and two strains of mice. We found a prominent sex difference in the currents during the first postnatal month when males had significantly greater nicotinic currents in layer VI neurons compared to females. These differences were apparent with three agonists: acetylcholine, carbachol, and nicotine. Furthermore, the developmental sex difference in nicotinic currents occurred despite male and female rodents displaying a similar pattern and proportion of layer VI neurons possessing a key nicotinic receptor subunit.

**Conclusions/Significance:**

This is the first illustration at a cellular level that prefrontal attention circuitry is differently affected by nicotinic receptor stimulation in males and females during development. This transient sex difference may help to define the cellular and circuit mechanisms that underlie vulnerability to attention deficit disorder.

## Introduction

Attention deficit disorders are at least twice as prevalent in males than females [1]–[3], yet the neurobiology behind this sex difference is not well understood. The normal development of the prefrontal cortex is critical for executive functions including attentional control [4]–[6]. Children with attention disorders appear to have higher activation of the prefrontal cortex at baseline and less change in its activation and synchronization with other cortical regions during the performance of attention tasks [7]. Within the prefrontal cortex, the corticothalamic neurons of layer VI are thought to play a key role in this cortical synchronization and also play a role in the thalamic gating necessary for attention [8]. However, very little is known about sex differences in the development of layer VI.

Recent work has shown that layer VI corticothalamic neurons in *male* rats are prominently excited by nicotinic acetylcholine receptors during early postnatal development [9]. This time period is developmentally equivalent to the last trimester of human gestation [10], [11]. Importantly, during this time, the prefrontal cortex is highly vulnerable to toxins and developmental insults [5], which predispose individuals to subsequent attention disorders. For example, prenatal exposure to the drug nicotine increases the risk of attention deficits [12], [13], particularly in males [14]. Interestingly, polymorphisms in the α4 nicotinic receptor subunit found in layer VI corticothalamic neurons have been associated with differences in performance on attention tasks [15]–[17]. However, most of these studies have not compared attentional performance by sex.

It is not known whether there are sex differences in the modulation of layer VI neurons by nicotinic acetylcholine receptors during development since previous work only examined male rats [9]. Here, we address this question with whole cell recording in acute brain slices of rodent prefrontal cortex across early postnatal development in both sexes. This technique allows us to assess the function of nicotinic receptors on layer VI pyramidal neurons and the effects of nicotine on these cells, without the confound that would arise *in vivo* due to different rates of systemic metabolism for nicotine in male and female rodents [18], [19].

## Materials and Methods

### Animals

These protocols conformed to international guidelines on the ethical use of rodents and were approved by the University of Toronto Animal Care and Use Committee. The founding mice were from Jackson Laboratory (Bar Harbor ME) and the rats from Charles River (Senneville PQ). Average litter sizes were 5–7 (mice) and 8–10 (rats). The pups were housed with their mothers until postnatal (P) day 21–22 and then housed in groups of 2–4 per cage. The facility has an ambient temperature of 22°C with a 12-hr light/dark cycle (lights on at 7 a.m.), and the cages have the following dimensions: (mouse) 7 ¾×12×6 ½” and (rat) 10 ½×19×8”.

### Brain Slice Preparation

After anaesthesia with choral hydrate (400 mg/kg), we prepared 400 µm thick coronal slices of the medial prefrontal cortex from male and female FVB mice (P7-P34), C57Bl/6 mice (P7-P28), and Sprague-Dawley rats (P14–28). The brain was cooled as rapidly as possible with 4°C oxygenated sucrose artificial cerebrospinal fluid (ACSF) with 254 mM sucrose substituted for NaCl. Prefrontal slices were cut from anterior to posterior using the appearance of white matter and the corpus callosum as anterior and posterior guides to target recording to the Cg1, Cg2 and PrL regions [20].

The slices were cut on a Dosaka Linear Slicer (SciMedia, Costa Mesa CA) and were transferred to room temperature oxygenated ACSF (128 mM NaCl, 10 mM D-glucose, 24 mM NaHCO_3_, 2 mM CaCl_2_, 2 mM MgSO_4_, 3 mM KCl, 1.25 mM NaH_2_PO_4_; pH 7.4) in a prechamber (Warner Instruments, Hamden CT) and allowed to recover for at least 1 hr prior to the beginning of an experiment. For whole cell recording, slices were placed in a modified superfusion chamber (Warner Instruments, Hamden CT) mounted on the stage of an Olympus BX50WI microscope (Olympus Canada, Markham ON). Regular ACSF at room temperature was bubbled with 95% oxygen and 5% carbon dioxide and flowed over the slice at 3–4 ml/minute.

### Electrophysiology

Whole cell patch electrodes (2–3 MΩ) contained 120 mM potassium gluconate, 5 mM KCl, 2 mM MgCl, 4 mM K_2_-ATP, 0.4 mM Na_2_-GTP, 10 mM Na_2_-phosphocreatine, and 10 mM HEPES buffer (adjusted to pH 7.33 with KOH). Medial prefrontal cortex layer VI neurons were patched under visual control using infrared differential interference contrast microscopy. In voltage-clamp, neurons were held at −75 mV, near the equilibrium potential for chloride under our conditions, and currents were recorded using continuous single electrode voltage clamp mode with an EPC10 (HEKA Electronics, Mahone Bay NS), acquired and low-pass filtered at 3 kHz with Patchmaster 2.20 (HEKA Electronics, Mahone Bay NS).

### Pharmacology

For most experiments, nicotinic currents were probed by adding 1 mM acetylcholine to the bath perfusion for a 15 s or 30 s interval, followed by a five-minute washout period. This concentration elicited a near-maximal response in both males and females that could be repeated reliably following a 5-minute washout period. Recordings were performed in the presence of atropine (200 nM) to block muscarinic receptors and methyllycaconitine (MLA; 10 nM) to block α7 nicotinic receptors. The peak current was measured in Clampfit (Molecular Devices) by subtracting the mean inward current at the peak (averaged over 1 s) of the acetylcholine response from the mean holding current during baseline (averaged over 30 s). The following compounds were added to the bath in specific experiments: 3 µM dihydro-β-erythroidine hydrobromide (DHβE), 1 mM carbachol, and 300 nM nicotine hydrogen tartrate. All compounds were obtained from Sigma (Sigma Aldrich Canada, Oakville ON) or Tocris (Cedarlane Laboratories, Burlington ON) and stored in stock solutions at −20°C before being diluted and applied to the slice in oxygenated ACSF.

### Immunohistochemistry

A knock-in mouse line in which the nicotinic acetylcholine receptor α4 subunit has been labeled with the YFP motif has been generated on a C57Bl/6 background and described previously [21]. Immunohistochemistry for YFP was performed to identify the distribution pattern of neurons containing 〈4 subunits in layer VI of male and female medial prefrontal cortex. Mouse brains were collected and 400 µm thick coronal sections of the prefrontal cortex were made as described above for electrophysiology. For each mouse, immunohistochemistry for YFP was performed on a brain slice that was directly anterior to the corpus callosum, corresponding with approximately Bregma +1.34 mm to +1.74 mm [20]. Slices were incubated in oxygenated ACSF for one hr and were then fixed in a solution containing 4% (wt/vol) paraformaldehyde in 100 mM phosphate buffer (pH 7.5) overnight at 4°C.

Free-floating sections were washed with Tris-buffered saline (TBS, 100 mM Tris and 150 mM NaCl, pH 7.5) and then incubated in 10% (wt/vol) bovine serum albumin (BSA), 0.25% (vol/vol) Triton X-100 and 4 drops/mL of a streptavidin solution (Vector Laboratories, Burlington ON) in TBS for 1 hr at room temperature. Sections were washed with TBS and incubated with a rabbit anti-GFP primary antibody which also recognizes YFP (Invitrogen, Burlington ON; 1∶200 dilution) with 3% (wt/vol) BSA, 0.25% (vol/vol) Triton X-100 and 4 drops/mL of a biotin solution (Vector Laboratories) in TBS for 72 hr at 4°C. Sections were washed in TBS and incubated with a biotinylated goat anti-rabbit secondary antibody (Invitrogen; 1∶500 dilution) with 3% (wt/vol) BSA and 0.25% (vol/vol) Triton X-100 in TBS for 24 hr at 4°C. Sections were washed in TBS and then incubated with streptavidin labeled with Alexa Fluor 594 (Invitrogen, 1∶500 dilution) with 3% (wt/vol) BSA and 0.25% (vol/vol) Triton X-100 in TBS for 24 hr at 4°C. Sections were washed with TBS, incubated in a solution containing 1.5 µg/mL 4',6-diamidino-2-phenylindole (DAPI) dilactate (Sigma Aldrich) in TBS for 2 hr at room temperature, washed again with TBS, mounted onto microscope slides and cover-slipped using Fluoromount G (SouthernBiotech, Birmingham AL).

### Imaging

Multi-photon imaging of the immunostained sections was performed using a Ti:sapphire laser (Mai Tai, Spectra Physics, Mountain View CA) tuned to wavelength 780 nm and an Olympus Fluoview FV1000 microscope (Olympus, Markham ON) with an Olympus XLPlan N 25x, 1.05 NA water-immersion objective. The inherent Z-sectioning in multiphoton imaging allowed us to examine the immunostaining in the top 20 µm of the slice where there was excellent penetration of the antibodies and DAPI. Green and red fluorescence were separated with a dichroic mirror at 570 nm and filtered with green: BA495–540 nm and red: BA570-625 nm filters (Olympus), respectively. Multiphoton images containing green and red channels, measuring 500 µm×500 µm (x,y), taken at equivalent depths from the top of the slice (approximately 12 µm deep), and having overlapping edges were captured with Olympus Fluoview FV10-ASW software. Six images were acquired per mouse covering the prelimbic and infralimbic areas of the medial prefrontal cortex from the pial surface to the white matter basal to layer VI. These images were then were stitched together to create one montage image using Image-Pro Plus software (Media Cybernetics, Bethesda, MD).

The proportion of neurons expressing the α4β2* nicotinic receptor was measured by counting the total number of YFP-immunoreactive neurons within a defined counting area of medial prefrontal layer VI in the red-channel montage and dividing by the total number of DAPI-positive neurons within that same counting area in the green-channel montage. Since DAPI can stain all cells, DAPI-positive neuronal nuclei were identified by the following criteria: their round shape with a diameter ≥7 µm and generally diffuse staining with a few discrete regions of intense staining that likely represent heterochromatin [22]. We found these criteria allowed us to differentiate between neuronal nuclei and those from endothelial cells (long and thin nuclei) and glia (smaller nuclei with intense DAPI staining). The use of similar criteria has been verified previously for use to identify neurons [22]. Further, we confirmed these criteria by staining slices with both DAPI and the neuronal-specific fluorescent Nissl stain NeuroTrace (1∶100, Invitrogen), as illustrated in the example in **Figure S1**.

The counting area was defined on the red-channel montages by first drawing a 750 µm-long basal line along the base of medial prefrontal layer VI (between layer VI and white matter) that generally extended along the base of the prelimbic and infralimbic areas. Next, a radial line was drawn at each end of the basal line that was perpendicular to the basal line and extended towards the pial surface, ending at the medial edge of the band of YFP-immunoreactive neurons. Last, a medial arc was drawn connecting the medial ends of the two radial lines and defining the curved medial length of the band of YFP-immunoreactive neurons. No measure showed a significant sex difference, but in accordance with stereological conventions, we only report the ratio of YFP-positive to DAPI-positive neurons.

### Statistical Analysis

We used parametric or non-parametric statistical tests when the data under analysis passed or failed respectively the Shapiro-Wilk test for normality. The developmental changes in male and female nicotinic currents were assessed with Kruskal-Wallis nonparametric ANOVA and *post hoc* Mann-Whitney nonparametric *t* tests. In order to test for gender and drug effects, DHβE, carbachol, and nicotine data were analyzed with two-way repeated measures ANOVA. *Post hoc* tests were performed to determine specific differences, when overall ANOVA results indicated significant effects of drug. Differences in acetylcholine response after nicotine exposure were determined using Wilcoxon signed-rank nonparametric paired *t* tests. Differences in intrinsic cell properties were examined with unpaired Student *t* tests. In all tests, a level of *P*<0.05 was required to indicate a significant difference. All data are expressed as the mean ± standard error.

## Results

### Developmental Differences in Nicotinic Currents in Male and Female FVB Mice

**Figure 1:**
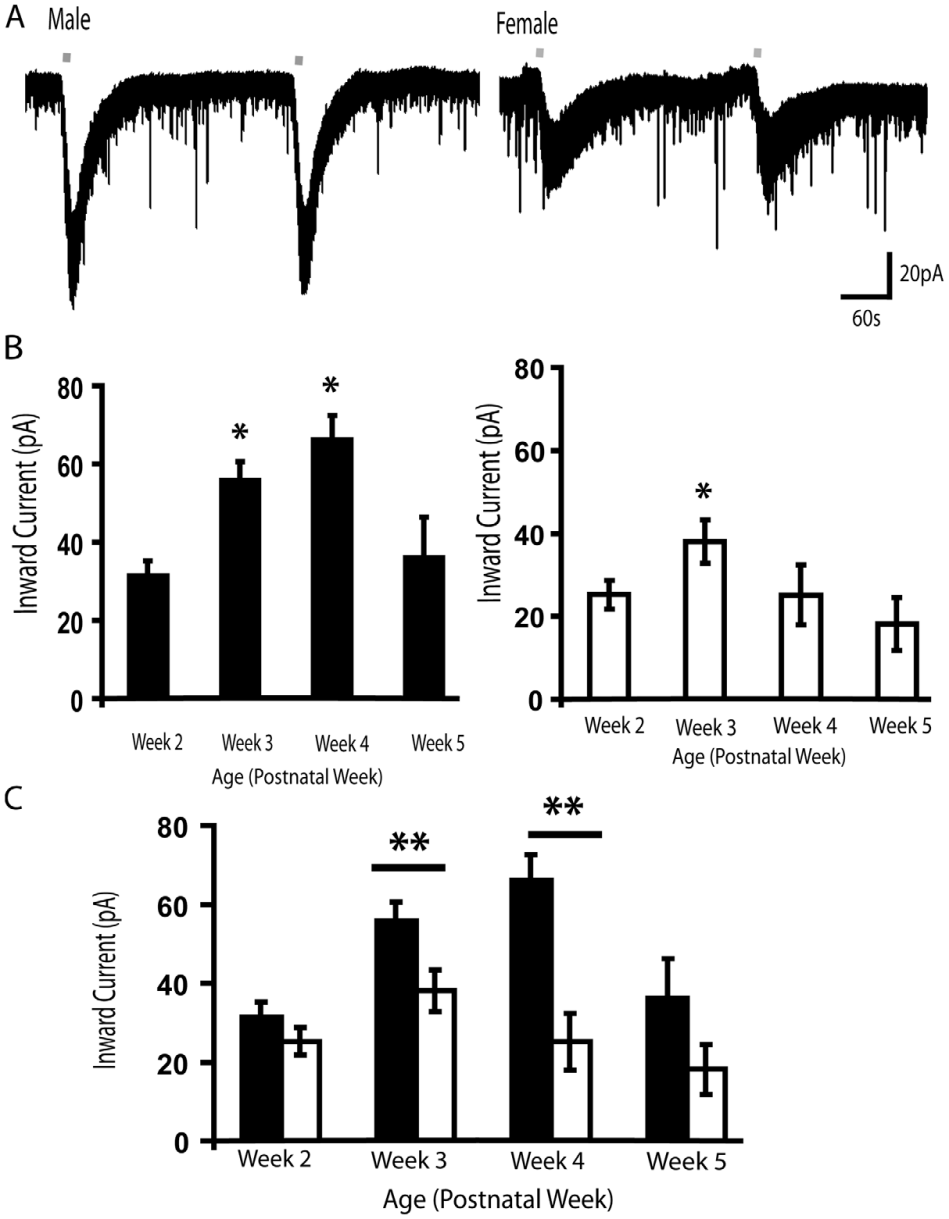
Developmental sex difference in nicotinic currents in layer VI neurons of prefrontal cortex. (**A**) Examples of voltage clamp traces from a P19 male and a P27 female showing nicotinic inward currents during bath application of acetylcholine (1 mM, 10 s). Line denotes acetylcholine application. Both males and females have reproducible, non-desensitizing currents elicited by bath-applied acetylcholine, when given five-minute washout duration. (**B**) Bar chart summarizing the mean amplitude of the peak inward current elicited by acetylcholine in FVB male (left panel) and female (right panel) mice in layer VI across postnatal weeks two to five. In males, there is a significant developmental effect where the mean nicotinic current during postnatal weeks three and four are significantly higher than the mean inward current during postnatal weeks two and five (* *P*<0.05). In females, there is also a significant developmental effect where the mean nicotinic current during postnatal week three is significantly higher than the mean inward current during postnatal weeks two and five (* *P*<0.05). (**C**) Bar graph displays the sex difference in the average inward current elicited by nicotinic receptor stimulation by acetylcholine (1 mM, 30 s). Males (black bars) have significantly greater currents than females (open bars) during postnatal weeks three and four (** *P*<0.01). All recordings are performed in the presence of atropine (200 nM) to block muscarinic receptors and MLA (10 nM) to block α_7_ nicotinic receptors.

We find that layer VI pyramidal neurons in mouse prefrontal cortex are excited by nicotinic acetylcholine receptors. To observe and characterize the sex differences in nicotinic currents, we performed whole cell recordings at −75 mV in brain slices from male and female mice. The nicotinic inward currents were stimulated by bath application of acetylcholine (1 mM, 30 s) in the presence of atropine (200 nM) to block muscarinic receptors, the G-protein-coupled subtype of acetylcholine receptors. Atropine is included in all subsequent experiments. Bath application of acetylcholine elicited inward currents in layer VI neurons as demonstrated in **Figure 1A** in males and females. Preliminary concentration-response analysis (100 µM–3 mM acetylcholine) within individual neurons suggested that 1 mM acetylcholine elicited near-maximal responses in both males and females, which could be reproduced following a five-minute washout period. Rapid, local application of acetylcholine can elicit currents of similar amplitudes to those obtained with bath application in layer VI pyramidal neurons [9] but, in fact, often elicits smaller currents since the placement of the applicator may preclude the stimulation of nicotinic receptors away from the soma. The size and reliability of the peak response with the bath application of 1 mM acetylcholine makes it an ideal measure to compare across different pharmacological conditions to assess the properties of the layer VI nicotinic currents.

In FVB mice, nicotinic inward currents in layer VI neurons showed significant developmental regulation as shown by Kruskal-Wallis ANOVA. Comparing the mean peak current amplitude across early postnatal weeks demonstrates the developmental upregulation of nicotinic excitation in male and female layer VI neurons as illustrated in **Figure 1B**. In both sexes there appears to be a developmental peak level of nicotinic currents (weeks three and four for males, week three for females) which declines significantly by week five; however, the developmental upregulation of the nicotinic currents appears less prominent in the female FVB mice. Further examination suggests that the week five nicotinic currents in layer VI pyramidal neurons are not significantly different to those in the adult FVB mice (adult males: 41±9 pA, n = 20; adult females: 39±6 pA, n = 24; unpaired *t* test, *P* = NS).

### Sex Differences in the Peak Amplitude of Nicotinic Currents in FVB Mice during Development

We observed a significant sex difference in the amplitude of the nicotinic currents elicited in layer VI neurons during the first postnatal month. As illustrated in **Figure 1C**, there is a sex difference in acetylcholine-elicited nicotinic inward currents during postnatal weeks three and four. Two-way ANOVA showed a significant effect of sex (F_1,241_ = 21.44, *P*<0.0001) and postnatal week (F_3,241_ = 5.79, P<0.001) on nicotinic currents and a significant interaction between sex and postnatal week (F_3,241_ = 2.68, *P*<0.05). Layer VI neurons from males had significantly higher currents than those from females during postnatal week three (males: 56±5 pA, *n* = 41; females: 38±5 pA, *n* = 43; Mann-Whitney test, *P*<0.01). Similarly, layer VI neurons from males had significantly higher currents than those from females during postnatal week four (males: 66±6 pA, *n* = 44; females: 25±7 pA, *n* = 22; Mann-Whitney test, *P*<0.01). During these weeks, there was no significant sex difference in the resting membrane potential (males: −77.4±2.6 mV; females: −77.1±2.4 mV; unpaired *t* test, *P* = NS) or input resistance (males: 246±11 MΩ, females: 272±12 MΩ; *P* = NS). Spike amplitude was slightly greater in neurons from females than males during postnatal weeks three and four (males: 87.5±5.4 mV, females: 94.4±2.5 mV, p<0.05). In order to look at nicotinic effects on excitability of layer VI pyramidal neurons of males and females during this developmental period, we applied acetylcholine (1 mM, 30 s) to a subset of neurons while recording in current clamp. Acetylcholine had significantly greater effects on excitability (Chi-squared test, *P*<0.05) in the male slices, where 77% (10 of 13 neurons) depolarized sufficiently to fire action potentials, compared to the female slices, where only 36% (5 of 14 neurons) depolarized to this extent.

### Layer VI Nicotinic Currents Mediated by α4β2* Nicotinic Receptors in Both Males and Females

To test our hypothesis that the nicotinic currents in layer VI neurons are mediated by α4β2* nicotinic receptors, we investigated the effects of the competitive antagonist di-hydro-β-erythroidine (DHβE) on the currents elicited by acetylcholine. We found that DHβE (3 µM, 10 min) almost completely suppressed the nicotinic currents in all layer VI neurons tested in both males and females. Two-way repeated measures ANOVA revealed a highly significant effect of DHβE (F_1,9_ = 122.21, *P*<0.0001) and no significant interaction between sex and the effects of DHβE on nicotinic currents. Male currents were significantly suppressed (control: 92±9 pA, DHβE: 19±5 pA; *n* = 6; paired *t* test; *P*<0.001; age-range examined: P16–P22). Female currents were also significantly suppressed (control: 63±9 pA; DHβE: 8±3 pA, *n* = 5; paired *t* test; *P*<0.01; age-range examined: P16–P23). This pharmacological data suggests that DHβE-sensitive receptors are the primary contributors to the nicotinic currents in layer VI pyramidal neurons in both male and female FVB mice. Residual current in the presence of DHβE was likely the result of competitive displacement of the antagonist by the high concentration of the agonist. Briefer application of acetylcholine (1 mM, 15 s) resulted in a current of similar amplitude to that elicited by the longer application, and DHβE completely eliminated the current in both sexes (n = 5; data not shown).

### Sex Differences in Nicotinic Currents Across Mouse Strains and Species of Rodent

To test if the developmental sex difference in nicotinic currents occurs across different strains of mice, we performed whole cell recordings on layer VI neurons from C57Bl/6 mice from weeks 2 to 4. Consistent with data from FVB mice, we found a sex difference in C57Bl/6 mice. Layer VI neurons from males had significantly greater inward currents elicited by acetylcholine than those from females during postnatal week three (males: 61±9 pA, *n* = 18; females: 32±7 pA, *n* = 17; Mann-Whitney test, *P*<0.05). Interestingly, the developmental upregulation of the nicotinic currents was restricted to week three in the male C57Bl/6 mice; however, this developmental upregulation appeared to be absent in the females.

To test if this developmental sex difference occurs across different rodent species, we examined the nicotinic currents in layer VI neurons of male and female rats from postnatal weeks 3 and 4. We chose this time period for analysis since it is consistent with the development peak for nicotinic currents in male rats [9]. Consistent with data from both strains of mice, we found a sex difference in rats. Layer VI neurons from male rats had significantly greater inward currents elicited by acetylcholine than female rats during postnatal week three (males: 80±13 pA, *n* = 25; females: 41±10 pA, *n* = 16; Mann-Whitney test, *P*<0.001). Similarly, males had greater inward acetylcholine-elicited currents than females during postnatal week four (males: 76±9 pA, n = 21; females: 38±7 pA, *n* = 11; Mann-Whitney test, *P*<0.01). The male rat data from weeks 3 and 4 is completely consistent with the means observed during the peak of the developmental upregulation during this time period in our previous study [9].

Thus, the developmental sex differences in layer VI nicotinic currents are observed in FVB and C57Bl/6 mice, and Sprague Dawley rats, where male rodents have significantly greater inward currents elicited by acetylcholine than female rodents during an important period of cortical development.

### A Prominent Sex Difference in the Nicotinic Currents Elicited by Carbachol, an Analogue of Acetylcholine Not Broken Down by Acetylcholinesterase

**Figure 2:**
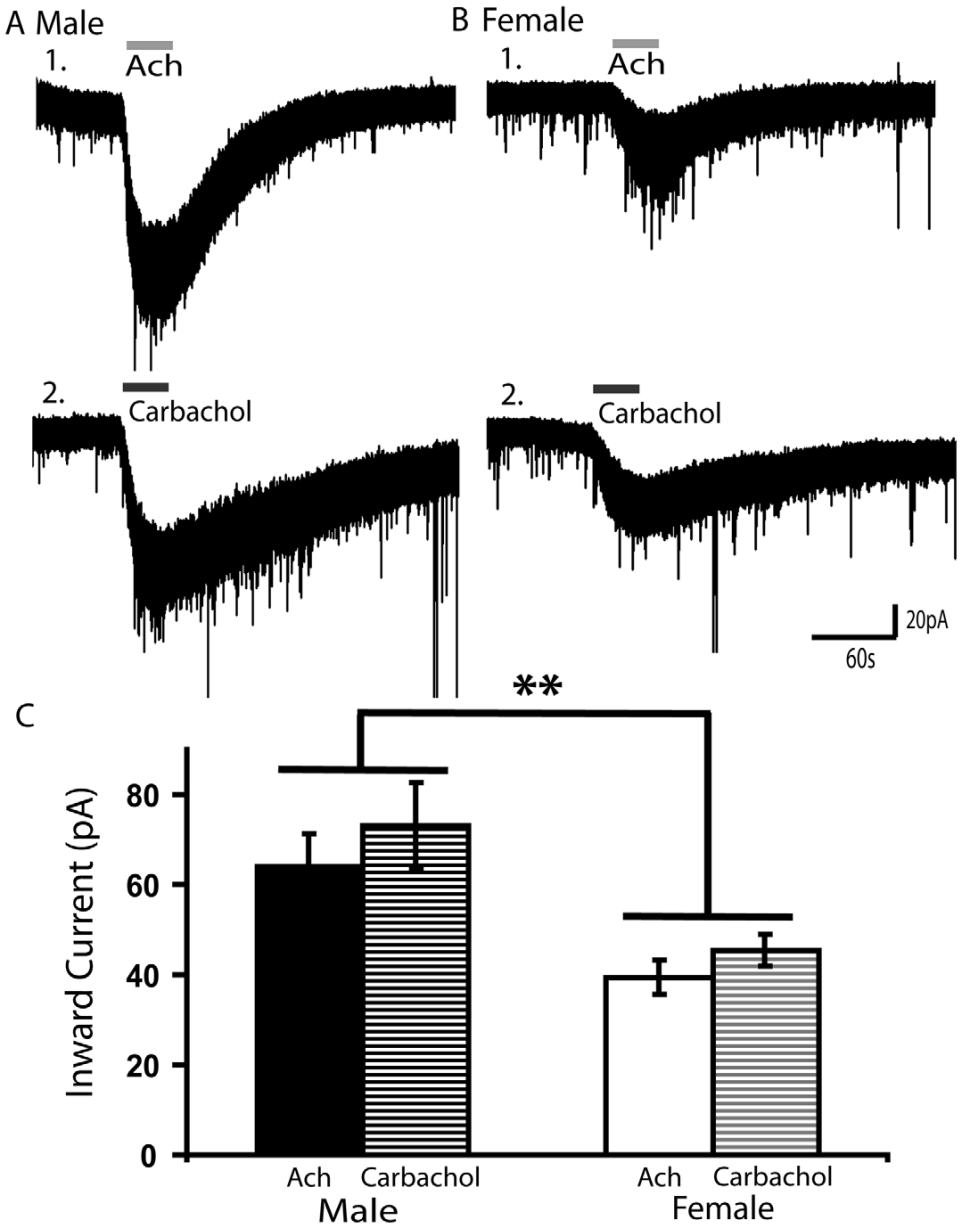
Developmental sex difference in nicotinic currents is *not* explained by different levels of acetylcholinesterase activity. (**A**) Voltage clamp traces showing inward currents during bath application of nicotinic acetylcholine receptor agonists (***1***) acetylcholine (1 mM, 30 s) and (***2***) carbachol (1 mM, 30 s), an acetylcholine analogue that is not broken down by endogenous acetylcholinesterase, in the same layer VI neuron from a P17 male FVB mouse. (**B**) Voltage clamp traces from the same agonist applications in a layer VI neuron from a P17 female FVB mouse. In both, males and females, the inward current persists longer after carbachol compared to acetylcholine, since the acetylcholinesterase in the brain slice metabolizes applied acetylcholine allowing the cell to return to baseline faster. (**C**) Bar chart summarizing the mean current amplitude elicited by 30 s application of 1 mM acetylcholine or carbachol (**P<0.01). The sex difference persists when the inward currents are elicited with 1 mM carbachol, suggesting that acetylcholinesterase levels do not account for the sex difference in nicotinic currents.

Acetylcholinesterase, the enzyme which metabolizes acetylcholine, is expressed in the deep layers of cingulate cortex early in postnatal development [23]. Sex differences in acetylcholinesterase activity have been previously reported in the cerebral cortex of adult rodents [24], suggesting that nicotinic currents elicited by acetylcholine might be under differential control by acetylcholinesterase in males and females during early postnatal development. To test whether different acetylcholinesterase activity accounts for the sex differences in nicotinic currents, we probed nicotinic currents using carbachol, a nicotinic receptor agonist that is not broken down by endogenous acetylcholinesterase. As expected, the inward currents elicited by carbachol (1 mM, 30 s) persisted for a longer duration compared to the inward currents elicited by acetylcholine (1 mM, 30 s) in both males and females, as seen in the voltage clamp traces in **Figure 2A and 2B**. This longer decay suggests that acetylcholinesterase normally contributes to the rapid removal of acetylcholine from the slice during the washout period. However, the peak current elicited by carbachol was very similar to that elicited by acetylcholine in both males (*n* = 12) and females (*n* = 13), as shown in **Figure 2C**. Two-way repeated measures ANOVA demonstrates a significant effect of sex (F_1,23_ = 11.59, *P*< 0.01), but no difference between acetylcholine and carbachol and no significant interaction between the effects of carbachol and sex. These results suggest that different levels of expression or activity of acetylcholinesterase do not account for our observed sex differences in nicotinic currents during development.

### Nicotine Elicits a Greater Inward Current in Males Compared to Females, but Similar Subsequent Desensitization of Acetylcholine Currents

**Figure 3:**
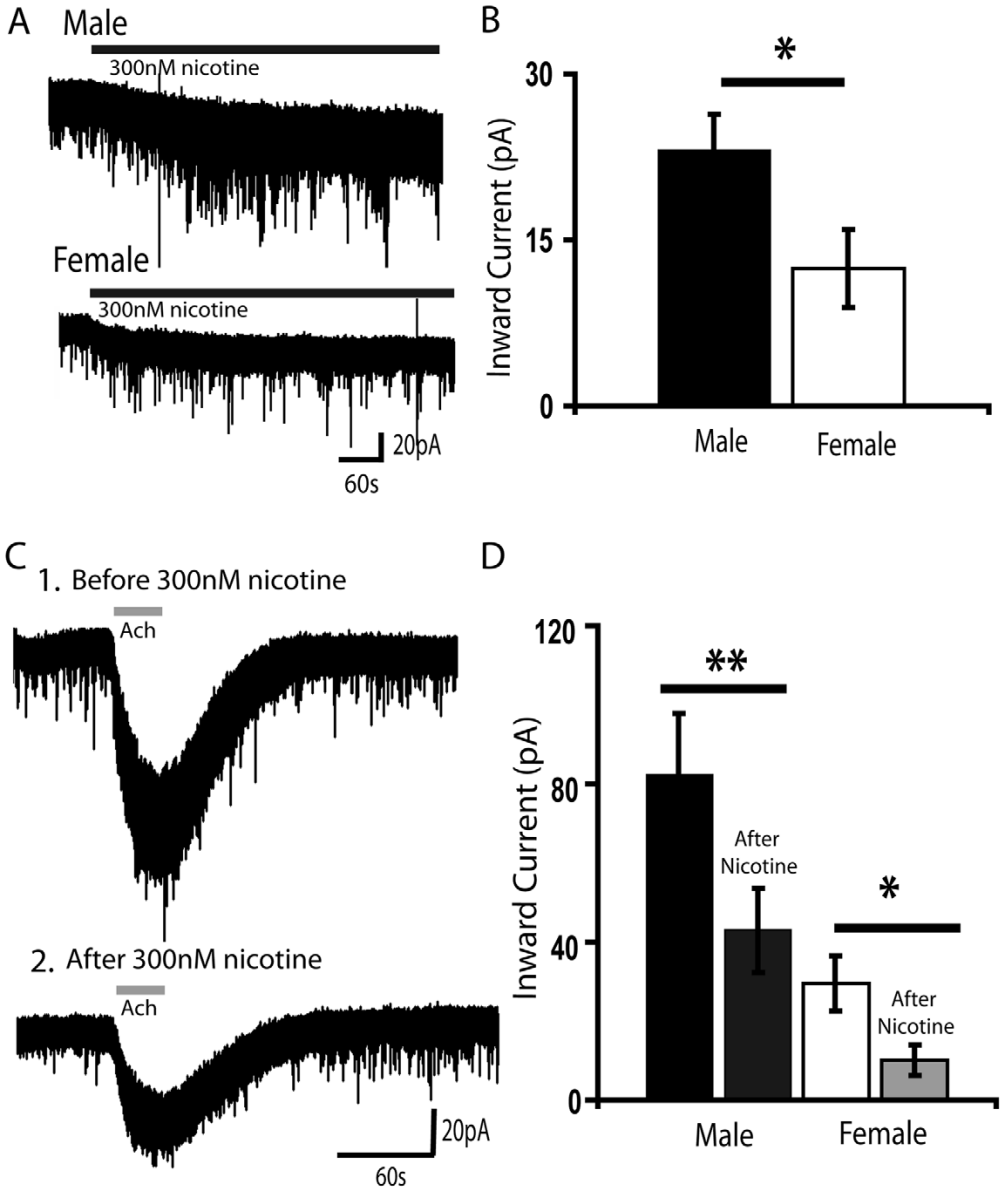
Developmental sex difference in current elicited by nicotine, but not its desensitization of acetylcholine currents. (**A**) Exemplary voltage-clamp traces showing a small, persistent inward current elicited by nicotine (300 nM, 10 min) in a layer VI neuron from a P21 male (top) and a P19 female (bottom). This concentration of nicotine is consistent with the peak blood level of nicotine seen in smokers [24] and is relevant to developmental nicotine exposure [25]. (**B**) Bar graph to the right showing the mean inward current elicited by 300 nM nicotine in typical male and female layer VI neurons. Nicotine elicited greater inward currents in male neurons than females (*P*<0.05). (**C** ***1***) A voltage-clamp trace from a P21 male shows a robust inward current with acetylcholine (1 mM, 30 s) before application of nicotine. (**C** ***2***) A voltage-clamp trace from the same neuron taken five minutes after the end of a ten minute application of nicotine (300 nM) shows that the inward current elicited by acetylcholine (1 mM, 30 s) is significantly decreased. (**D**) Bar chart showing the highly significant suppression of the inward current elicited by acetylcholine (1 mM, 30 s) in males and females after the above nicotine exposure. The latter inward currents elicited by acetylcholine were examined five minutes after nicotine application when its inward current had returned to baseline (*P<0.05, ** *P*<0.01).

A concentration of nicotine (300 nM), consistent with the peak blood level seen in smokers [25], elicited a larger inward current in layer VI neurons from male FVB mice than in those from females. The voltage clamp traces in **Figure 3A** illustrate the persistent inward currents elicited by nicotine (300 nM, 10 min) in male (top) and female (bottom) layer VI neurons. The bar chart illustrated in **Figure 3B** shows the mean currents elicited by nicotine in male and female layer VI neurons. The inward current elicited by nicotine is greater in layer VI neurons from males than females in the third and fourth postnatal weeks: (males: 23±3 pA, *n* = 11; females: 12±4 pA, *n* = 8; unpaired *t* test, *P*<0.05). These results with a relatively low concentration of nicotine are consistent with the sex difference observed with the near maximal nicotinic receptor stimulation with acetylcholine and carbachol.

We then investigated the extent of nicotine-induced desensitization of the currents elicited by acetylcholine in male and female layer VI neurons. At the time that the inward current elicited by nicotine had returned to baseline (∼5 minutes washout; [9]), the subsequent inward current in response to acetylcholine was suppressed in both male and female FVB mice. Two-way repeated measures ANOVA demonstrates a significant effect of sex (F_1,13_ = 5.16, *P*< 0.05), an extremely significant effect of nicotine desensitization (F_1,13_ = 37.12, *P*<0.0001) and no significant interaction between nicotine desensitization and sex. **Figure 3C** illustrates a representative response to acetylcholine before and after a ten-minute application of nicotine, showing a significant suppression of the current elicited by acetylcholine. **Figure 3D** shows the mean inward currents before and after nicotine in males and females: (males: 82±16 pA before, 43±11 pA after, n = 10; Wilcoxon signed rank test, *P*<0.01, age range examined: P14–P26; females: 29±7 pA before and 10±4 pA after, n = 5; Wilcoxon signed rank test *P*<0.05, age range examined: P15–P26). Thus, while a concentration of nicotine that is relevant to developmental nicotine exposure [25], [26] is able to activate larger inward currents in male layer VI neurons than females, this exposure substantially desensitizes the nicotinic currents elicited by acetylcholine in both male and female mice.

### Similar Proportion of Layer VI Neurons with the α4 Nicotinic Subunit in Males and Females

**Figure 4:**
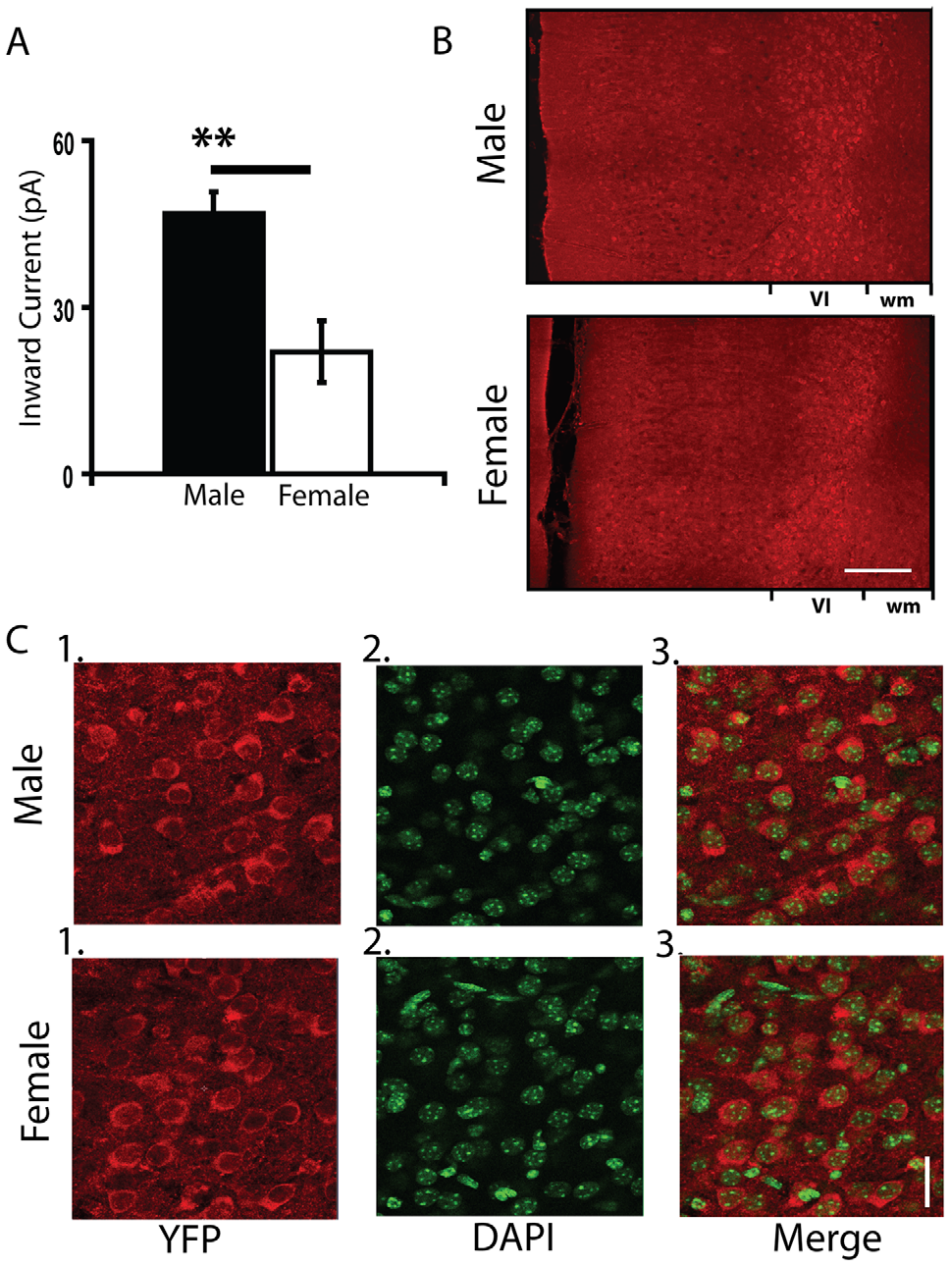
Developmental nicotinic currents and nicotinic α4YFP-positive neurons in male and female knock-in mice. (**A**) Bar graph showing larger inward currents in male α4YFP knock-in mice compared to age-matched female α4YFP knock-in mice during postnatal week three (**P<0.01). (**B**) Low-magnification image of P15 male and female prefrontal cortex slices with the YFP signal on the α4YFP subunits amplified using a 3-step immunohistochemistry protocol described in the Methods section. Both sexes show a distinct neuronal band of staining in layer VI of the medial prefrontal cortex (bright red cells), showing the presence of α4* nicotinic receptors. Scale bar: 200 µm. (**C**) High-magnification of (***1***) YFP immunostained neurons, (***2***) DAPI stained cells, and (***3***) merged images within layer VI of male (top images) and female (bottom images) prefrontal cortex. The criteria for identifying DAPI-positive neurons are described in the Methods section. The proportion of neurons expressing α4YFP was not significantly different between males and females. Scale bar: 20 µm.

During postnatal weeks three and four, 96% (82 of 85 neurons) of male but only 83% (54 of 65 neurons; Chi-squared test, *P*<0.01) of female layer VI neurons showed an inward current elicited by acetylcholine which was greater than 3x RMS baseline noise. While there remained a significant sex difference in the amplitude of the currents after removing the non/minimal responders (males: 63±4 pA, n = 82; females: 41±5 pA, n = 54; unpaired *t* test, *P*<0.001), the lower proportion of cells responsive to acetylcholine suggested that male and female mice may have a different proportion of layer VI neurons containing nicotinic receptors. To address this question, we examined layer VI neurons in a C57Bl/6 strain of knock-in mice expressing fluorescent α4* nicotinic receptors (α4YFP; [21]). By homologous recombination in these mice, YFP was inserted in the gene encoding for the M3-M4 cytoplasmic domain of the α4 nicotinic receptor subunit rendering a fluorescently tagged α4 subunit. As demonstrated in **Figure 4A**, electrophysiological examination of prefrontal brain slices from these mice show nicotinic currents in layer VI neurons with a prominent sex difference at postnatal week three: (males: 57±6 pA, *n* = 34; females: 28±7 pA, *n* = 15; Mann Whitney test, *P*<0.01). This difference is similar to what we recorded in the wildtype C57Bl/6 mice in the previous section. Furthermore, 100% (34 of 34 neurons) of male and only 80% (12 of 15 neurons) of female layer VI neurons in these α4YFP mice showed inward currents in response to acetylcholine (Chi-squared test, *P*<0.05). Therefore, this knock-in mouse is a suitable model for studying possible anatomical substrates of our observed sex differences.

To study the distribution pattern and proportion of layer VI cells that express nicotinic receptors, we amplified the YFP signal with a 3-step immunohistochemistry protocol (detailed in Materials and Methods) in P15–16 mice. As demonstrated in **Figure 4B**, prefrontal cortex slices from male and female mice show a prominent labeling of YFP-positive cells (shown in red) in layer VI. To detect differences in the proportion of nicotinic receptor-expressing cells in layer VI, we compared the number of cells expressing α4YFP to neuronal nuclei labeled by DAPI (shown in green, see Methods for criteria to determine neurons labeled by DAPI and **Figure S1** for staining with DAPI and NeuroTrace). **Figure 4C** shows high-magnification images of α4YFP in layer VI neurons in male and female prefrontal cortex respectively. The same areas are shown stained for DAPI, in addition to the merged images of YFP immunostaining and DAPI. The ratio of α4YFP-expressing to DAPI-stained cells was not significantly different in males and females (males: 0.76±0.02, *n* = 5 mice; females: 0.73±0.03, *n* = 5 mice; unpaired *t* test, *P* = NS). While we cannot distinguish between receptors inserted in the cell membrane and those in intracellular compartments, this data suggests that males and females do not differ in the pattern or proportion of neurons positive for α4YFP in layer VI of prefrontal cortex during development.

## Discussion

In this study, we found a prominent developmental sex difference in the nicotinic currents activated by acetylcholine in layer VI pyramidal neurons of the prefrontal cortex. The specific 〈4®2* nicotinic receptor antagonist, DH®E, suppressed these currents in both sexes suggesting the currents are mediated predominantly by 〈4®2* nicotinic acetylcholine receptors. The sex difference persisted when the nicotinic receptors were activated with carbachol, an analogue of acetylcholine that is not broken down by endogenous acetylcholinesterase. Consistent with this data, nicotine applied at the peak concentration of nicotine found in the blood of smokers [25] produced larger inward currents in male layer VI neurons when compared to female layer VI neurons. The prominent sex differences in nicotinic excitation seen with several different agonists prompted an anatomical investigation of nicotinic receptors in layer VI neurons during early postnatal development. We used a knock-in line of 〈4YFP mice and found a sex difference in the nicotinic currents in layer VI but no difference in the proportion of YFP-positive neurons between males and females. Together, our data raise important questions about the mechanism that underlies the prominent sex difference in functional nicotinic currents during development as well as the consequences of this sex difference for the maturation of corticothalamic attention circuitry.

### Potential Mechanisms Underlying Sex Differences in Developmental Nicotinic Currents

The functional sex differences in nicotinic currents we observed could arise at several potential levels. The maturation process of nicotinic receptors involves a highly-regulated assembly process in the endoplasmic reticulum, followed by the trafficking of the receptor to the membrane [27]. Sex and developmental differences in these processes have not been extensively examined. Developmental sex differences have not been reported in the expression of any rodent nicotinic subunits, including the cortical α4 and β2 subunits which form the majority of nicotinic receptors in layer VI [28], [29], nor the α5 subunit [29], [30] which may act as an accessory subunit in these neurons [9]. As illustrated in our immunohistochemical analysis and described previously, there is a large intracellular population of nicotinic subunits [21], [27]. In fact, a large percentage of the assembled receptors may also be located intracellularly [27], [31] and therefore would not contribute to the electrophysiologically-recorded currents. One chaperone molecule that increases the surface expression of 〈4®2* nicotinic receptors [32] has been shown to be developmentally regulated in primary culture of cortical neurons [33]. There may also be developmental and sex differences in the length of time nicotinic receptors remain in the membrane before being subject to ubiquitylation, a process involved in nicotinic receptor degradation [34].

On the other hand, the differences in nicotinic currents in male and female rodents may result from sex differences in cortical neurosteroid levels either directly or through differential nuclear translocation of steroid receptors. The sex steroid progesterone has been reported to influence nicotinic receptor function directly through negative allosteric modulation of 〈4®2* nicotinic receptors [35], [36]. The sex differences we observe in the present study occur during the pre-pubertal period, before the surge of gonadal hormones. However, the rodent brain expresses all the enzymes necessary for the *de-novo* synthesis of progesterone from cholesterol [37], [38], and the rate limiting enzyme in this pathway shows a trend toward greater cortical expression in females than males at P10 [37]. Interestingly, progestin binding is concentrated in layer VI cortical region early in development [39], [40], suggesting that progesterone receptors are expressed in the deep layers of cortex. In agreement with the timing of the sex differences we found in nicotinic currents in the present study, male and female differences in nuclear progesterone receptor binding are evident at postnatal days 14 and 21, with females having significantly greater nuclear translocation of progesterone receptors [41] than age-matched males.

### Consequences of Developmental Sex Differences in Nicotinic Stimulation of Layer VI Neurons

Lesions of the cholinergic system during development alter cortical circuitry [42], [43] and neuronal morphology [44], [45], with implications for attention. It is likely that the developmental excitation of high-affinity nicotinic receptors by endogenous acetylcholine during this period of development would influence synaptic plasticity, as has recently been shown with excitation of nicotinic receptors in prefrontal interneurons [46]. In the case of layer VI pyramidal neurons, the timing of the sex differences in nicotinic currents suggests that they may contribute to sex differences in the refinement and maturation of cortical projections to the inhibitory thalamic reticular neurons and excitatory thalamic projection neurons [8], [47], [48], [49]. These projections control the coordination of excitation and inhibition of the thalamus that underlies attention [8]. The maturation of corticothalamic circuitry is influenced by nicotinic receptors during development [50], [51] and thus may contribute to lifelong sex differences in tasks involving attention [52].

Prenatal nicotine exposure is strongly associated with an increased incidence of attention deficit disorders [13], [53]. Sex differences in the effects of developmental nicotine exposure on brain and behavior have been reported in rodents and humans [14], [18], [54], [55]. Work by Jacobsen showed that males exposed to nicotine during gestation had the most severe impairment in auditory attention tasks [14]. However, these findings are confounded by potential sex differences in the systemic metabolism of nicotine. In rodents and humans, nicotine is metabolized at different rates in males and females [19], [56] and thus, may be present at different concentrations in the brain in males and females [57]. An advantage of our experimental approach is that slice electrophysiology allows nicotinic receptor agonists and modulators to be applied directly to the brain slice at known concentrations and under controlled pharmacological conditions.

Under certain circumstances, it is possible that greater nicotinic excitation of layer VI neurons in males during cortical maturation may lead to a greater number of stabilized synapses and thus a higher baseline activation of prefrontal cortex, a pattern which has been observed in human imaging studies of ADHD [7]. Increased distractibility is a prominent feature of ADHD that can result from the inability of the prefrontal cortex to sufficiently deactivate with increasing difficulty of attention tasks [7]. It is important for future studies to examine how nicotinic excitation of layer VI neurons affects their innervation and activation of both inhibitory and excitatory thalamic nuclei.

## Supporting Information

Figure S1 An example multiphoton merged image of layer VI cells which are stained with both DAPI (green) and NeuroTrace (red). Since DAPI can label cells other than neurons, we used the following criteria to count a DAPI-positive cell as a neuron: round shape with a diameter ≥7 µm, generally diffuse staining with punctate regions of intense staining that likely represent heterochromatin [22]. Here, we show that the DAPI nuclei that meet these criteria are also co-labeled by NeuroTrace. By contrast, the red arrows illustrate example DAPI nuclei that do not meet the neuronal criteria due to their shape, size, and/or intensity of staining. The latter cells were not co-labeled by NeuroTrace. Scale bar: 50 µm.
(0.66 MB JPG)
