# Supplementary figures and images for: Developmental Sex Differences in Nicotinic Currents of Prefrontal Layer VI Neurons in Mice and Rats
Source: PLoS One. 2010 Feb 17;5(2):e9261. doi: 10.1371/journal.pone.0009261 (PMC2822857; doi:10.1371/journal.pone.0009261)

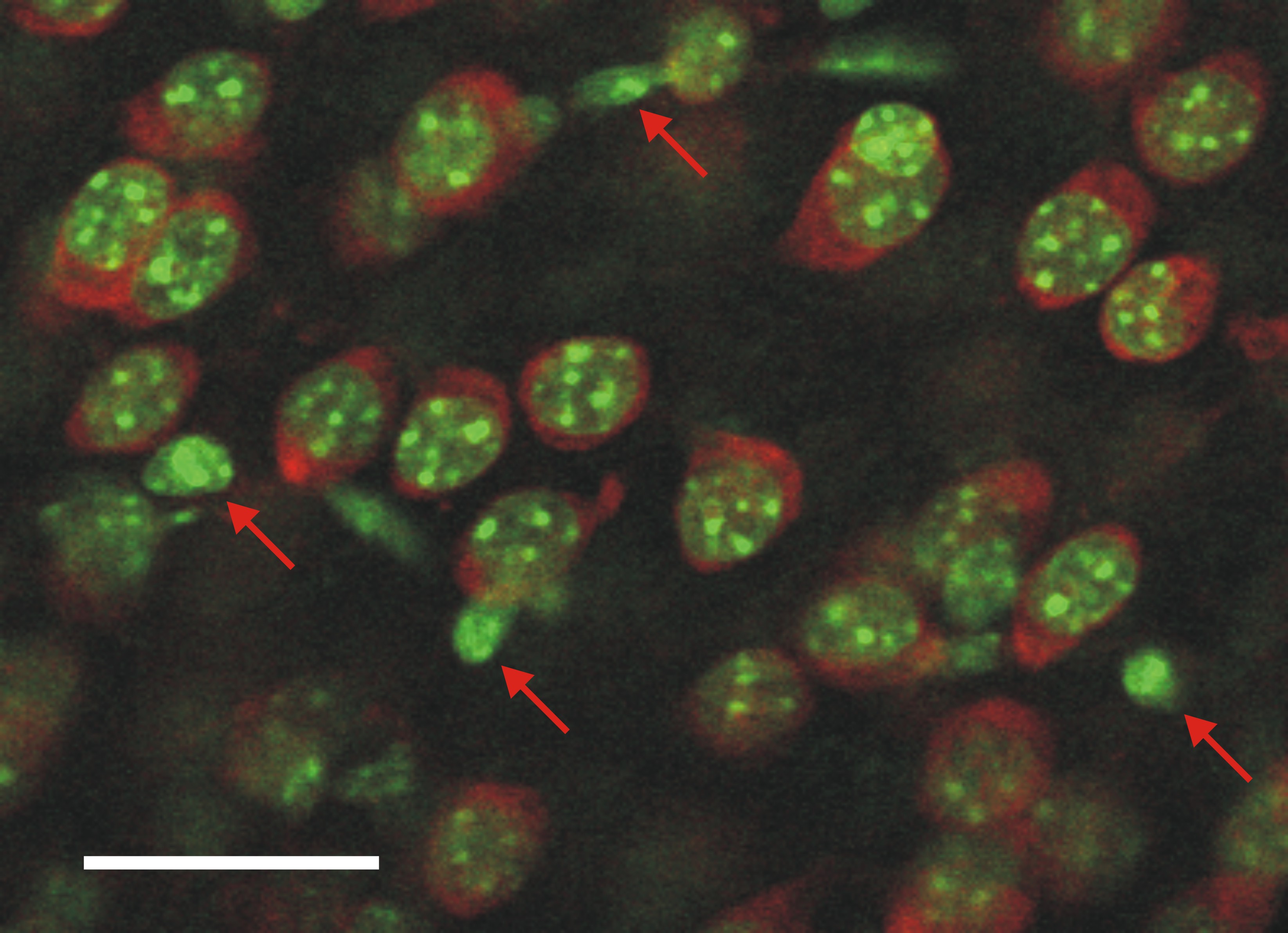

Supplement: Figure S1 — An example multiphoton merged image of layer VI cells which are stained with both DAPI (green) and NeuroTrace (red). Since DAPI can label cells other than neurons, we used the following criteria to count a DAPI-positive cell as a neuron: round shape with a diameter ≥7 µm, generally diffuse staining with punctate regions of intense staining that likely represent heterochromatin [22]. Here, we show that the DAPI nuclei that meet these criteria are also co-labeled by NeuroTrace. By contrast, the red arrows illustrate example DAPI nuclei that do not meet the neuronal criteria due to their shape, size, and/or intensity of staining. The latter cells were not co-labeled by NeuroTrace. Scale bar: 50 µm. (0.66 MB JPG) [file pone.0009261.s001.jpg]
